# Intra-arterial tenecteplase is safe and may improve the first-pass recanalization for acute ischemic stroke with large-artery atherosclerosis: the BRETIS-TNK trial

**DOI:** 10.3389/fneur.2023.1155269

**Published:** 2023-04-18

**Authors:** Zi-Ai Zhao, Jing Qiu, Lu Wang, Yong-Gang Zhao, Xian-Hui Sun, Wei Li, Xin Liu, Xiao-Long Li, Liang Liu, Ming-Rui Chen, Hui-Sheng Chen

**Affiliations:** Department of Neurology, General Hospital of Northern Theater Command, Shenyang, China

**Keywords:** tenecteplase, endovascular treatment, first-pass reperfusion, ischemic stroke, large vessel occlusion

## Abstract

**Background and purpose:**

The first-pass recanalization of endovascular treatment (EVT) is closely correlated with clinical outcome of patients with large vessel occlusion (LVO) stroke. The aim of the study was to explore whether intra-arterial tenecteplase (TNK) during the first pass of EVT can increase first-pass successful reperfusion and improve the neurological outcome in AIS-LVO patients.

**Materials and methods:**

The BRETIS-TNK trial (ClinicalTrials.gov Identifier: NCT04202458) was a prospective, single-arm, single center study. Twenty-six eligible AIS-LVO patients with large-artery atherosclerosis etiology were consecutively enrolled from December 2019 to November 2021. Intra-arterial TNK (4 mg) after microcatheter navigation through the clot was administered, followed by TNK (0.4  mg/min) given continuously for 20  min after the first retrieval attempt of EVT without confirmation of the reperfusion status by DSA. The 50 control patients comprised of a historical cohort before the BRETIS-TNK trial (from March 2015 to November 2019). Successful reperfusion was defined as modified Thrombolysis In Cerebral Infarction (mTICI) ≥2b.

**Results:**

The first-pass successful reperfusion rate was higher in the BRETIS-TNK vs. control group (53.8% vs. 36%, *p* = 0.14), and the difference became statistically significant after propensity score matching (53.8% vs. 23.1%, *p* = 0.03). There was no difference in symptomatic intracranial hemorrhage between the BRETIS-TNK and control groups (7.7% vs. 10.0%, *p* = 0.92). There was a trend toward higher proportion of functional independence at 90 days in the BRETIS-TNK comparing with the control group (50% vs. 32%, *p* = 0.11).

**Conclusion:**

This is the first study to report that intra-arterial TNK during the first pass of EVT seems safe and feasible in AIS-LVO patients.

## 1. Introduction

Acute ischemic stroke (AIS) is one of the most important causes of neurological morbidity and mortality in the world, especially in patients with large vessel occlusion (LVO) (1). Endovascular treatment (EVT) has been approved as the most effective treatment for AIS-LVO patients, but not all patients receiving EVT have good outcomes (2–6). It has been reported that only 46% of AIS-LVO patients who undergo successful reperfusion after EVT achieved functional independence at 90 days (7). First-pass successful reperfusion is widely accepted to be associated with favorable outcome (8–10). In addition, a high number of device passes are associated with worse outcome (11, 12), often attributed to increased clot fragmentation with distal embolization and/or endothelial damage after several retrieval attempts. Moreover, poor outcome in patients may be attributed to a no reflow phenomenon, with impaired microvascular reperfusion despite complete angiographic reperfusion (13, 14).

During EVT for AIS-LVO patients with large-artery atherosclerosis (LAA) etiology, additional retrieval attempts may be needed to achieve recanalization, which may result in endothelial damage and microvascular impairment, hence decreasing the likelihood of successful reperfusion (15, 16). A growing body of literature has shown that tenecteplase (TNK) was similar or superior to alteplase for the treatment of LVO AIS due to higher fibrin specificity, greater resistance to inhibitors of tissue plasminogen activator, and longer half-life (17). For example, the EXTEND-IA TNK trial demonstrated that intravenous TNK at 0.25 mg/kg significantly improved reperfusion and clinical outcomes compared with alteplase (18). Recent CHOICE trial indicated that among AIS-LVO patients with successful reperfusion following thrombectomy, the use of adjunct intra-arterial alteplase compared with placebo resulted in a greater likelihood of excellent neurological outcome at 90 days (19).

In this context, we hypothesized that intra-arterial TNK during the first attempt of EVT may increase the rate of first-pass successful reperfusion and improve the neurological outcome in AIS-LVO patients with LAA etiology. The current study was designed to explore the safety, feasibility, and possible efficacy of the combination in this population.

## 2. Materials and methods

### 2.1. Study design and participants

The BRETIS-TNK study (NCT 04202458) was a prospective, single-arm, single center study in which AIS-LVO patients with LAA etiology according to TOAST classification (20) were treated with a combination of thrombectomy and intra-arterial TNK administration. The trial was approved by the ethics committee of the General Hospital of Northern Theatre Command. Written informed consents were obtained from all patients or their surrogates before EVT started. The study was performed in accordance with the ethical standards as laid down in the 1964 Declaration of Helsinki and its later amendments or comparable ethical standards. The inclusion criteria were: (1) age ≥ 18 years; (2) anterior circulation AIS-LVO patients who were eligible for EVT according to AHA/ASA guideline (21); (3) LVO due to probable LAA etiology confirmed by DSA and clinical and neuroimaging data; (4) availability of informed consent. Probable LAA etiology was supported by clinical findings (such as a history of neurological deficit fluctuation, transient ischemic attacks in the same vascular territory, and exclusion of major-risk sources of cardiogenic embolism such as atrial fibrillation), signs of atherosclerosis of the culprit artery (such as stenosis of large arteries with ulcer, calcifications indicated by CTA and DSA). The LAA etiology was further confirmed by other tests such as cardiac imaging, electrocardiogram, and laboratory assessments during the following hospitalization (20). The exclusion criteria were: (1) other sub-types of ischemic stroke such as cardioembolism; (2) hemorrhagic stroke; (3) coagulation disorders, tendency for systemic hemorrhage, or thrombocytopenia (<100,000/mm^3^); (4) severe hepatic or renal dysfunction (more than 2 times upper limit of normal value in ALT or AST; more than 1.5 times of upper limit of normal value in serum creatinine) or requiring dialysis; (5) severe uncontrolled hypertension (systolic blood pressure over 200 mmHg or diastolic blood pressure over 110 mmHg which was unable to be controlled); (6) patients allergic to any ingredient of drugs in our study; (7) unsuitable for the clinical study as assessed by the clinician. Intravenous thrombolysis was not excluded in this study. Due to non-randomized design, historical control patients who met the same inclusion/exclusion criteria before BRETIS-TNK trial beginning were screened from our EVT database and these data were collected between March 2015 and November 2019.

### 2.2. Procedures

**Figure 1:**
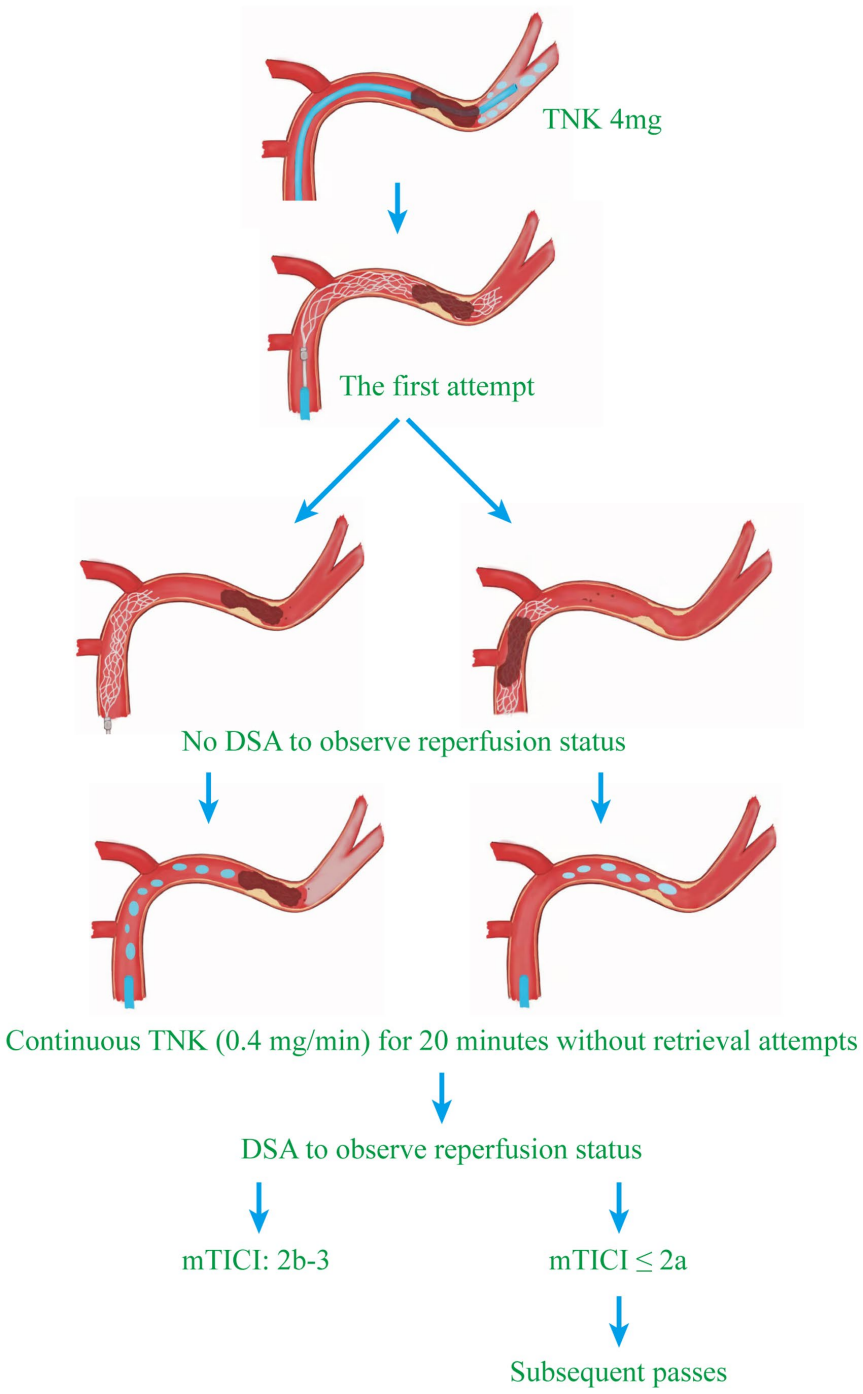
Flowchart of endovascular treatment procedures. The strategy of intra-arterial TNK administration in this study was composed of two steps. The first step was an infusion of 4  mg TNK after the microcatheter navigation across the clot. Then, a stent retriever device was deployed through the microcatheter from distal to proximal across the site of occlusion. The second step was an infusion of intra-arterial TNK (0.4  mg/min) continuously for 20 min after the first retrieval attempt. A DSA was performed to determine the status of the occluded vessel. If successful reperfusion was not achieved, subsequent passes were performed according to standard procedure.

EVT procedures were performed in a biplane neuroangiography suite (Philips UNIQ Clarity FD20/20; Philips, the Netherlands) under local anesthesia as described previously (22). Briefly, unfractionated heparin was infused at 50–100 IU/Kg at first and additional 1,000 IU at intervals of an hour during the operation (23). As shown in Figure 1, an infusion of 4 mg TNK (Guangzhou Recomgen Biotech Co., Ltd.) was administered through the microcatheter after microcatheter navigation across the clot and removal of the microwire. A stent retriever device (Solitaire FR) was then selected according to labeling indications and deployed through the microcatheter from distal to proximal across the site of occlusion. After slow withdrawal of the stent retriever and aspiration catheter, intra-arterial TNK (0.4 mg/min) was given continuously for 20 min via relocated aspiration catheter proximal to the site of occlusion after the first thrombectomy pass, and then followed by DSA to determine the status of the occluded vessel. Given that continuous TNK infusion (0.4 mg/min) will be obviously interrupted by the retrieval maneuver, no retrieval attempts were performed by interventionalists during the 20–30 min of TNK administration. If successful reperfusion was not achieved, then subsequent passes were performed according to standard procedure. Procedures in the historical group were performed following the same standard in the BRETIS-TNK group.

### 2.3. Outcomes

The primary outcome was the proportion of successful reperfusion defined as modified Thrombolysis In Cerebral Infarction (TICI) 2b-3 when continuous TNK infusion finished after the first-pass. The secondary outcomes were the proportion of final successful reperfusion, good clinical outcome (defined as modified Rankin Scale score, 0–2) at 90 days and early neurological improvement (ENI), which was defined as more than 4 point decrease in NIHSS within 24 h (24). The safety outcomes included symptomatic intracranial hemorrhage (sICH) within 48 h, which was defined as an increase in the NIHSS score of ≥4 points as a result of the intracranial hemorrhage (25), all-cause mortality within 90 days, and intracerebral hemorrhage, which was classified according to clinical and radiological criteria using the ECASS trial definitions of hemorrhagic infarction (HI1, HI2), parenchymal hemorrhage (PH1, PH2), and remote parenchymal hemorrhage (PHr1, PHr2) (26). Outcomes were assessed by neuroradiologists and clinicians blinded to the clinical data.

### 2.4. Statistical analysis

Data are expressed as the means with 95% confidence interval (CI), as medians with interquartile range (IQR), or absolute and relative frequency distribution, unless otherwise specified. Comparisons of baseline demographic, clinical, imaging, and procedural characteristics were evaluated by one-way analysis of variance on ranks followed by Dunn’s method for discontinuous or nonnormal variables or student t test followed by Bonferroni *post-hoc* test for continuous and normally distributed variables or chi-square tests. The primary and secondary outcomes were estimated using a binomial regression model adjusted for the admission NIHSS and onset to reperfusion time (ORT). Further, we performed a 1:1 propensity score matching based on the nearest-neighbor matching algorithm with a caliper width of 0.1 of the propensity score to reduce the possibility of selection bias in the historical control cohort. The propensity score was estimated using a logistic regression model adjusted for age, admission NIHSS, ORT, location of occlusion, and iv-thrombolysis, which may influence the successful reperfusion or functional outcome (7, 8, 27–29). No missing data imputation was conducted in this study due to no missing data for the baseline and outcomes. Statistical analyses were performed using SPSS version 26.0 (SPSS Software, IBM Corp., Armonk, NY, United States). A *p* value of 0.05 was considered statistically significant.

## 3. Results

**Figure 2:**
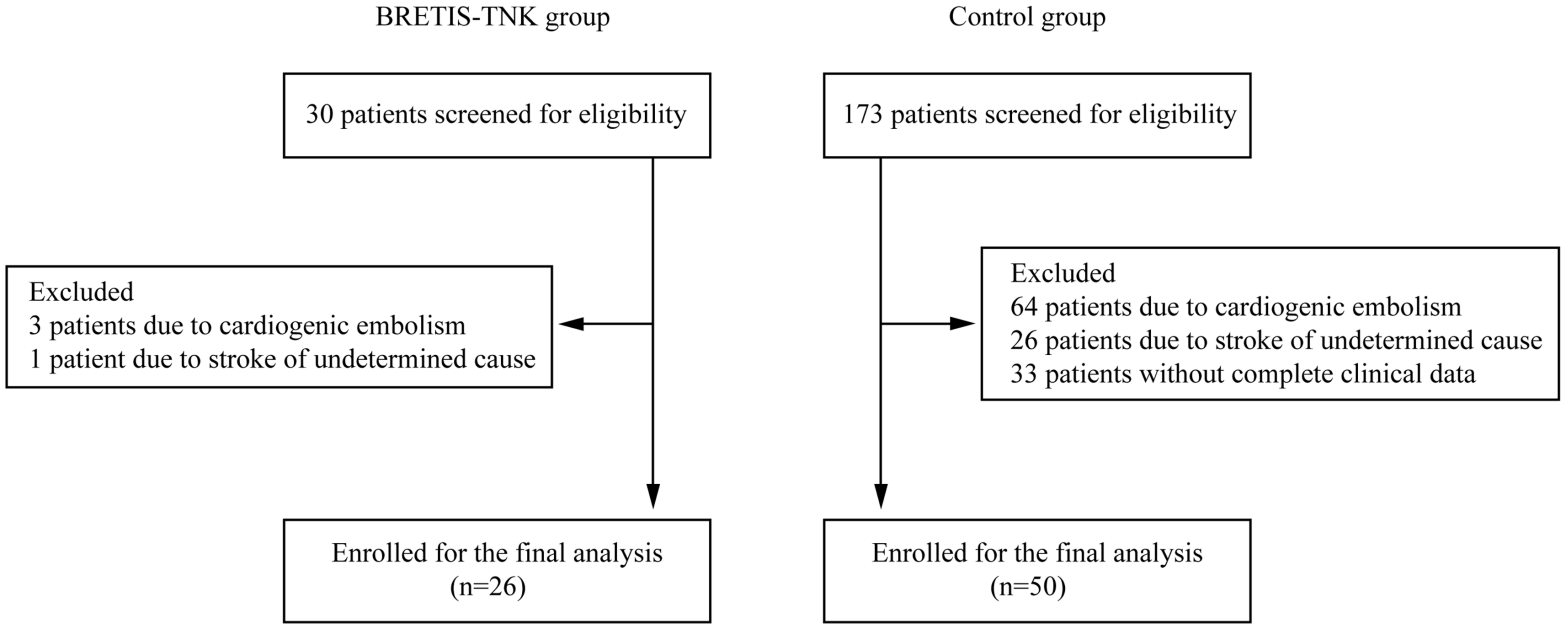
Flowchart of patient inclusion.

Between December 2019 and November 2021, 26 patients with LVO stroke who met inclusion criteria were enrolled in the BRETIS-TNK group and 50 patients were included in the historical control group after 173 patients were screened for eligibility (Figure 2). Baseline demographic, clinical, imaging, and interventional characteristics are comparable between the BRETIS-TNK vs. control group respectively: median age [61.5 (IQR 54–67.5) vs. 60 (IQR 54–65); *p* = 0.81], intravenous thrombolysis rate (26.9% vs. 18%; *p* = 0.37), premorbid mRS (*p* = 0.42), baseline NIHSS score (15.35 ± 4.18 vs. 15.44 ± 3.79; *p* = 0.92), last known normal to puncture time [414.0 (IQR 272.5–597.3) min vs. 438.5 (IQR 248.0–617.5) min; *p* = 0.97], percentage of MCA occlusions (61.5% vs. 56.0%; *p* = 0.64), ASPECTS at baseline [8 (IQR 7–10) vs. 9 (IQR 8–10); *p* = 0.61], the puncture to reperfusion time [89.5 (IQR 71.0–129.3) min vs. 88.0 (IQR 54.25–141.75) min; *p* = 0.59], total number of retrieval attempts [1 (IQR1-2) vs. 2 (IQR 1–2); *p* = 0.341], and thrombectomy techniques (direct aspiration or stent-retriever as the first attempt) (Table 1). Aspiration was performed as frontline to reduce the potential extracranial thrombus burden in 4 patients (8%) with tandem occlusions in the BRETIS-TNK group and 13 patients (26%) in the control group. Then, the intraarterial TNK administration and retrieval maneuver were performed to resolve the intracranial occlusion of terminal ICA or MCA, which were regarded as the first-pass retrieval attempts in this study.

**Table 1:** Demographic and clinical characteristics.

	BRETIS-TNK group (*n* = 26)	Control group (*n* = 50)	*p-*value
Age, year, median (IQR)	61.5 (54–67.5)	60 (54–65)	0.81
Sex (n, %)			0.77
Male	22 (84.6)	41 (82.0)	
Female	4 (15.4)	9 (18.0)	
Medical history (n, %)			
Hypertension	18 (69.2)	31 (62.0)	0.53
Diabetes	7 (26.9)	8 (16.0)	0.26
Ischemic stroke	8 (30.8)	22 (44.0)	0.24
Current smoker	20 (76.9)	31 (62.0)	0.19
Admission blood pressure Mean ± S.D			
SBP (mmHg)	140.08 ± 19.25	142.57 ± 21.78	0.81
DBP (mmHg)	82.73 ± 9.80	82.98 ± 13.21	0.81
Premorbid mRS (n, %)			0.42
0	24 (92.3)	43 (86.0)	
1	2 (7.7)	7 (14.0)	
Admission NIHSS, mean ± S.D	15.35 ± 4.18	15.44 ± 3.79	0.92
Baseline ASPECTS, median (IQR)	8 (7–10)	9 (8–10)	0.61
LKN to puncture time (min), median (IQR)	414.0 (272.5–597.3)	438.5 (248.0–617.5)	0.97
DPT (min), median (IQR)	64.5 (38.75–114.5)	49.5 (37.75–79.25)	0.10
PRT (min), median (IQR)	89.5 (71.0–129.3)	88.0 (54.25–141.75)	0.59
ORT (min), median (IQR)	582.81 (392.25–671)	505.5 (339.75–718.25)	0.67
Prior intravenous thrombolysis (n, %)^a^	7 (26.9)	9 (18.0)	0.37
Location of intracranial occlusion on angiography (n, %)			0.64
M1	16 (61.5)	28 (56.0)	
Terminal ICA	10 (38.5)	22 (44.0)	
Tandem occlusion	4 (8)	13 (26)	0.29
Ipsilateral cervical carotid occlusion (n, %)	5 (19.2)	13 (26)	0.51
Techniques of EVT			0.29
Aspiration catheter as frontline followed by stent retriever	4 (8)	13 (26)	
Stent retriever	22 (92)	37 (74)	
Additional bailout balloon dilation (n, %)	4 (15.4)	7 (14)	1
Additional bailout stenting (n, %)	3 (11.5)	3 (6)	0.406
Passes, median (IQR)	1 (1, 2)	2 (1, 2)	0.341

ASPECTS, Alberta Stroke Program Early CT Score; LKN, last known normal; DPT, Door-to-puncture time; PRT, puncture to reperfusion time; ORT, onset to reperfusion time.

^a^In 3 patients EVT was performed when intravenous thrombolysis was still running.

**Figure 3:**
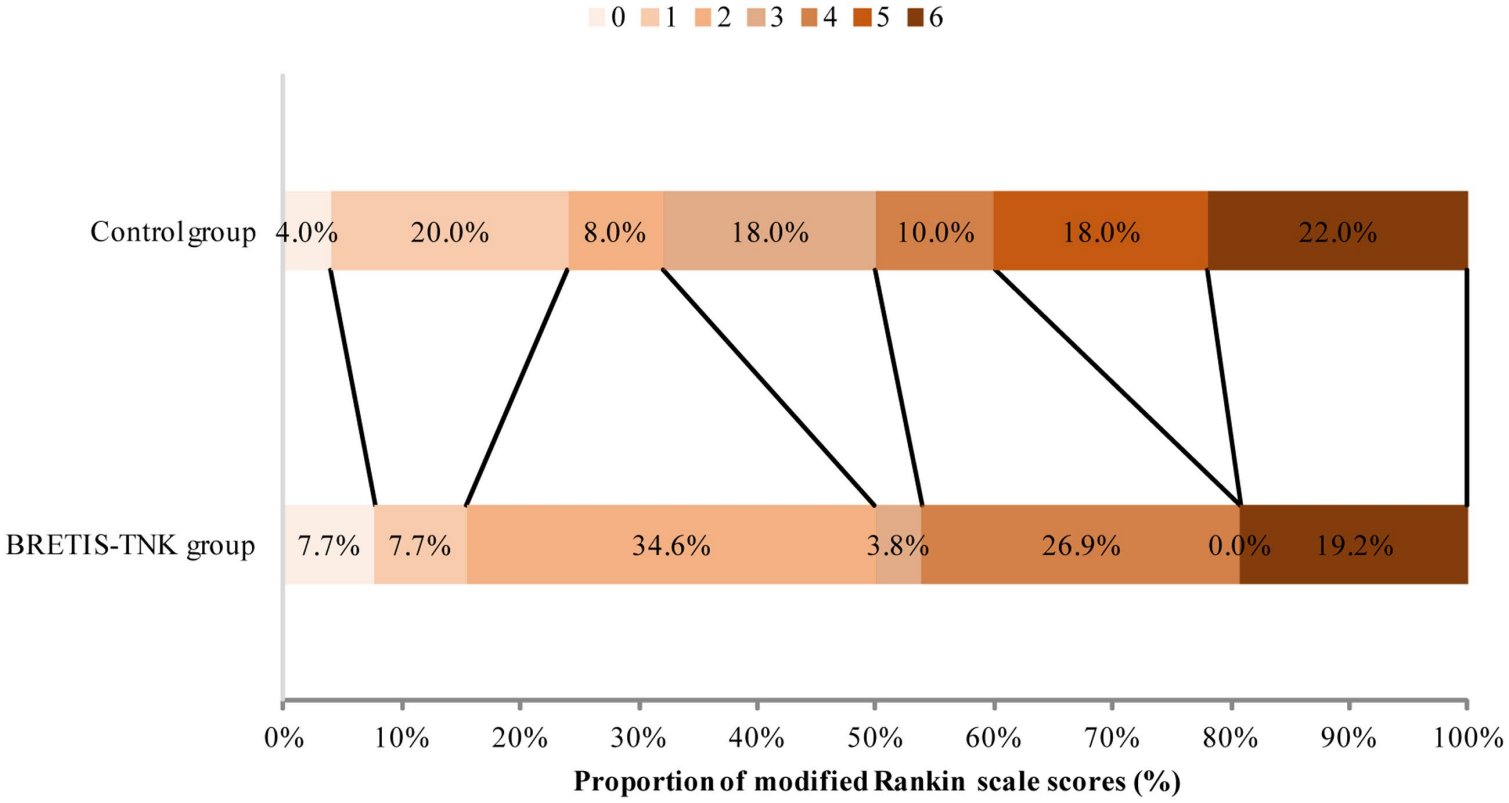
Distribution of functional scores at 90 days. Scores range from 0 to 6 (0 indicating no symptoms; 1, symptoms without clinically significant disability; 2, slight disability; 3, moderate disability; 4, moderately severe disability; 5, severe disability; 6, death). There was a trend toward higher proportion of functional independence at 90 days in the BRETIS-TNK comparing with the control group (50.0% vs. 32.0%, adjusted odds ratio, 2.32; 95% CI, 0.83–6.51; *p =* 0.11) although not statistically significant.

There was numerically higher first-pass successful reperfusion rate (53.8% vs. 36.0%; adjusted odds ratio, 2.10; 95% CI, 0.79–5.55; *p =* 0.14), proportion of patients with ENI (34.6% vs. 22.0%, adjusted odds ratio, 1.93; 95% CI, 0.67–5.58; *p* = 0.23), and good clinical outcome (50.0% vs. 32.0%, adjusted odds ratio, 2.32; 95% CI, 0.83–6.51; *p =* 0.11) in the BRETIS-TNK group vs. control group although not statistically significant (Table 2 and Figure 3). The final successful reperfusion rate was comparable between two groups (80.8% vs. 78.0%; adjusted odds ratio, 1.19; 95% CI, 0.36–3.87; *p* = 0.78). As for safety, there were comparable rates of intracranial hemorrhage including sICH between the two groups (Table 2). At 90 days, death occurred in 5 of 26 (19.2%) patients in the BRETIS-TNK group vs. 11 of 50 (22%) patients in the control group (adjusted odds ratio, 0.89; 95% CI, 0.26–3.10; *p* = 0.86) (Table 2).

**Table 2:** Outcomes.

	BRETIS-TNK group (*n* = 26)	Control group (*n* = 50)	Unadjusted		Adjusted	
			Odds ratio (95% CI)	*p-*value	Odds ratio (95% CI)	*p-*value
*Primary outcome*						
First-pass successful reperfusion (n, %)	14 (53.8)	18 (36.0)	2.07 (0.79–5.44)	0.14	2.10 (0.79–5.55)	0.14*
*Secondary outcomes*						
mRS (0–2) at 90 days (n, %)	13 (50.0)	16 (32.0)	2.13 (0.80–5.62)	0.13	2.32 (0.83–6.51)	0.11*
ENI	9 (34.6)	11 (22.0)	1.88 (0.66–5.36)	0.24	1.93 (0.67–5.58)	0.23*
*Safety outcome*						
sICH (n, %)	2 (7.7)	5 (10.0)	0.75 (0.14–4.16)	0.74	0.91 (0.14–5.89)	0.92*
*Other outcomes*						
Final successful reperfusion (n, %)	21 (80.8)	39 (78.0)	1.19 (0.36–3.87)	0.78	1.19 (0.36–3.87)	0.78*
mRS at 90 days, median (IQR)	2.5 (2–4)	3.5 (1.75–5)		0.42		
Any ICH (n, %)	8 (30.8)	19 (38.0)	0.73 (0.26–1.99)	0.53	0.73 (0.26–2.05)	0.55*
HI-1	1 (3.8)	1 (2.0)				
HI-2	0 (0.0)	5 (10.0)				
PH-1	1 (3.8)	1 (2.0)				
PH-2	6 (23.1)	11 (22.0)				
PHr-1	0 (0.0)	0 (0.0)				
PHr-2	0 (0.0)	1 (2.0)				
Death within 90d (n, %)	5 (19.2)	11 (22.0)	0.84 (0.26–2.76)	0.78	0.89 (0.26–3.10)	0.86*

mRS, modified Rankin Scale; ENI, early neurological improvement; sICH, symptomatic intracerebral hemorrhage; ICH, intracerebral hemorrhage; HI, hemorrhagic infarct; PH, parenchymal hemorrhage; PHr, remote parenchymal hemorrhage. *Adjusted for key prognostic covariates (NIHSS at admission and ORT).

As shown in Table 3, higher first-pass successful reperfusion rate was observed in the BRETIS-TNK group (53.8%) compared with the control group (23.1%) (adjusted odds ratio, 3.95%; 95% CI, 1.17–13.35; *p* = 0.03) after propensity score matching. The other outcomes were consistent with those before propensity score matching (Table 3).

**Table 3:** Outcomes after propensity score matching.

	BRETIS-TNK group (*n* = 26)	Control group (*n* = 26)	Unadjusted		Adjusted	
			Odds ratio (95% CI)	*p-*value	Odds ratio (95% CI)	*p-*value
*Primary outcome*						
First-pass successful reperfusion (n, %)	14 (53.8)	6 (23.1)	3.89 (1.18–12.84)	0.03	3.95 (1.17–13.35)	0.03*
*Secondary outcomes*						
mRS (0–2) at 90 days (n, %)	13 (50.0)	9 (34.6)	1.89 (0.62–5.76)	0.26	1.99 (0.60–6.57)	0.26*
ENI	9 (34.6)	6 (23.1)	1.77 (0.52–5.97)	0.36	1.89 (0.54–6.56)	0.32*
*Safety outcome*						
sICH (n, %)	2 (7.7)	2 (7.7)	1.00 (0.13–7.69)	1.00	2.362 (0.16–35.47)	0.53*
*Other outcomes*						
Final successful reperfusion (n, %)	21 (80.8)	19 (73.1)	1.55 (0.42–5.70)	0.51	1.61 (0.43–6.12)	0.48*
mRS at 90 days, median (IQR)	2.5 (2, 4)	3.5 (1, 5)		0.58		
Any ICH (n, %)	8 (30.8)	7 (26.9)	1.21 (0.36–4.01)	0.76	1.26 (0.37–4.33)	0.71*
HI-1	1 (3.8)	0 (0.0)				
HI-2	0 (0.0)	1 (3.8)				
PH-1	1 (3.8)	0 (0.0)				
PH-2	6 (23.1)	6 (23.1)				
PHr-1	0 (0.0)	0 (0.0)				
PHr-2	0 (0.0)	0 (0.0)				
Death within 90d (n, %)	5 (19.2)	5 (19.2)	1 (0.25–3.97)	1.00	1.34 (0.28–6.36)	0.71*

mRS, modified Rankin Scale; ENI, early neurological improvement; sICH, symptomatic intracerebral hemorrhage; ICH, intracerebral hemorrhage; HI, hemorrhagic infarct; PH, parenchymal hemorrhage; PHr, remote parenchymal hemorrhage. *Adjusted for key prognostic covariates (NIHSS at admission and ORT).

## 4. Discussion

Despite successful reperfusion, AIS-LVO patients treated with EVT frequently do not achieve a favorable outcome, otherwise known as futile reperfusion (27). Many factors and mechanisms have been attributed to futile reperfusion including poor collateral flow (30), multiple thrombectomy maneuvers (11, 12), blood vessel injury, reocclusion (29, 31), and impairment of microcirculatory reperfusion or the no-reflow phenomenon (13, 14). The first-pass successful reperfusion is an important determinant for good clinical outcome, because additional thrombectomy maneuvers may increase the risk of distal embolization and endothelial injury (8, 10, 32, 33), which may result in impairment of microcirculatory reperfusion after recanalization. In the present study, higher first-pass successful reperfusion rate as well as numerically higher proportion of patients with functional independence were found in the BRETIS-TNK group, compared with the control group, with similar rates of intracerebral hemorrhage. Collectively, the pilot study suggests that the combination strategy of intra-arterial TNK during the first attempt of thrombectomy seems safe and feasible, and may have the potential to increase first-pass reperfusion rates and the proportion of patients achieving functional independence.

We only enrolled AIS-LVO patients with LAA etiology in this study given the difficulty in achieving recanalization during EVT in AIS-LVO patients with LAA etiology (15, 16). Results from the Safe Implementation of Treatment in Stroke (SITS) thrombectomy register indicated a lower chance of reperfusion and worse outcomes after thrombectomy in patients with LAA compared to cardiac embolism etiology (34). Previous studies have found that intra-arterial thrombolysis can increase recanalization and improve clinical outcomes (35). However, data on the safety and efficacy of intra-arterial thrombolysis adjunct to mechanical thrombectomy are scarce. A systematic review and meta-analysis indicated no increase in the risk of sICH after intra-arterial fibrinolytics as adjunct to mechanical thrombectomy (36). Intra-arterial TNK was found to be safe with comparable favorable outcomes with alteplase and reteplase (37). BRETIS-TNK is the first attempt to determine the safety and efficacy of intra-arterial TNK during the first pass of EVT. There are several mechanisms by which intra-arterial TNK could exert its effect. First, intra-arterial thrombolysis may increase the efficiency of thrombectomy given that the thrombus will be loosened or lysed by the first pass, resulting in higher first-pass effect (35, 38). Second, intra-arterial thrombolysis before or after the first pass could dissolve the distally embolized thrombus and microthrombus, which may improve the microcirculation (39). Administration of intra-arterial thrombolytic was further supported by the recent CHOICE trial that among AIS-LVO patients with successful reperfusion following EVT, the use of adjunct intra-arterial alteplase compared with placebo resulted in a greater likelihood of excellent neurological outcome at 90 days (19). In the current study, no DSA was performed to measure the reperfusion status during intra-arterial infusion of TNK (0.4 mg/min) after the first retrieval maneuver, because the original idea was that if reperfusion was achieved after the first pass, the following TNK administration may exert the similar effect as alteplase in the CHOICE trail (19), while if reperfusion was not achieved after the first pass, the following TNK administration may rescue thrombolysis as recombinant prourokinase reported in PROACT II trail (35). Taken together, we contend that if intra-arterial TNK can partially improve on the angiographically visible circulation and microcirculation as mentioned above, the clinical outcome in AIS-LVO patients with LAA could be improved. Our current findings in this pilot study lend support to this hypothesis. The combined strategy of mechanical and/or pharmacological thrombolysis will be important to be confirmed in multi-center, double blinded, randomized control trials.

In the current study, TNK was chosen as the thrombolytic of choice based on recent findings that TNK may have better efficacy on clinical and imaging endpoints compared to alteplase, with good safety profile (40). The intra-arterial TNK strategy including the selected dose was empirical in the absence of related literature at the time of study design. Hence, the optimal intra-arterial TNK strategy, whether before or after successful reperfusion as in the CHOICE trial, is uncertain, and merits investigation in future trials. In the BRETIS-TNK group, the rate of sICH was 7.7%, which is comparable to the control group and previous reports (7). This pilot trial suggests the feasibility, safety and possible efficacy of the current strategy. In this trial, heparin was used during the procedure. Considering recent evidence that heparinization during EVT may be associated with increased risk of symptomatic intracranial hemorrhage (41), intravenous unfractionated heparin was not used in our BRETIS-TNK II trial (NCT05657444).

### Limitations

First, this trial was nonrandomized in design and lacked a contemporary control arm. Given the retrospective nature of control group, the confounding factors such as the changes in EVT procedures, and the improvement of technical capabilities as well as of stroke care with time may have affected the outcomes. In addition, multiple attempts were allowed without waiting 20 min in historical controls, which may shorten the procedural time and could affect outcome. Second, this is a single center study with a small sample, which could render our findings inconclusive. Third, higher rate of ICA occlusions (44%) was noted in the control group vs. 38.5% in the BRETIS-TNK group, which may have contributed to the low reperfusion rate and less favorable outcome in the control group. We included patients with both tandem occlusion and intracranial LAA, which may affect outcome given the difference in treatment effect between two groups. Finally, in the present study, an infusion of intra-arterial TNK (0.4 mg/min) via the relocated aspiration catheter was administrated no matter whether the first passage was successful or not. Based on this design, we did not perform DSA after removal of the stent retriever and aspiration catheter to minimize the potential adverse effects of contrast agent, which prevented us from distinguishing how many patients acquire recanalization due to the one thrombectomy maneuver or TNK administration. Given this limitation and the safety concern, different doses of TNK infusion will be used based on the status of vessel patency after retrieval maneuver in the BRTIS-TNK II (NCT05657444), aiming to further explore the efficacy and safety of intra-arterial TNK administration during EVT.

## 5. Conclusion

To our knowledge, this is the first report that intra-arterial TNK as adjunct to mechanical thrombectomy seems safe and feasible in AIS-LVO patients with LAA etiology. However, because of study limitations, these findings should be interpreted as preliminary and a prospective multicenter randomized trial is warranted.

## Data availability statement

Publicly available datasets were analyzed in this study. This data can be found at: The data that support the findings of this study are available from the corresponding author upon reasonable request.

## Ethics statement

The studies involving human participants were reviewed and approved by the Ethics Committee of the General Hospital of Northern Theatre Command. The patients/participants provided their written informed consent to participate in this study.

## Author contributions

Z-AZ, JQ, LW, X-HS, Y-GZ, WL, XL, X-LL, LL, M-RC, and H-SC contributed to the acquisition of the data and the drafting of the manuscript. Z-AZ, LW, and H-SC conducted data analysis. H-SC contributed to the study design and critically edited the manuscript. All authors contributed to the article and approved the submitted version.

## Funding

This work was supported by the Shenyang Science and Technology Bureau (20–205–4-007), the Science and Technology Project Plan of Liao Ning Province (2020JH1/10300002). H-SC received the fundings.

## Conflict of interest

The authors declare that the research was conducted in the absence of any commercial or financial relationships that could be construed as a potential conflict of interest.

## Publisher’s note

All claims expressed in this article are solely those of the authors and do not necessarily represent those of their affiliated organizations, or those of the publisher, the editors and the reviewers. Any product that may be evaluated in this article, or claim that may be made by its manufacturer, is not guaranteed or endorsed by the publisher.
